# Risk of COVID-19 death in cancer patients: an analysis from Guy’s Cancer Centre and King’s College Hospital in London

**DOI:** 10.1038/s41416-021-01500-z

**Published:** 2021-08-16

**Authors:** Beth Russell, Charlotte L. Moss, Vallari Shah, Thinzar Ko Ko, Kieran Palmer, Rushan Sylva, Gincy George, Maria J. Monroy-Iglesias, Piers Patten, Muhammed Mansour Ceesay, Reuben Benjamin, Victoria Potter, Antonio Pagliuca, Sophie Papa, Sheeba Irshad, Paul Ross, James Spicer, Shahram Kordasti, Danielle Crawley, Harriet Wylie, Fidelma Cahill, Anna Haire, Kamarul Zaki, Ailsa Sita-Lumsden, Debra Josephs, Deborah Enting, Angela Swampillai, Elinor Sawyer, Andrea D’Souza, Simon Gomberg, Claire Harrison, Paul Fields, David Wrench, Anne Rigg, Richard Sullivan, Austin Kulasekararaj, Guy’s Cancer Real World Evidence, Eleanor Jones, Pavetha Seeva, Christina Karampera, Aarani Devi, Fareen Rahman, Daniel Smith, Kasia Owczarczyk, Eirini Tsotra, Charalampos Gousis, Mary Lei, Sharmistha Ghosh, George Nintos, Kavita Raj, Mary Gleeson, Katherine Bailey, Richard Dillon, Matthew Streetly, Anca Mera, Jasmine Timbres, Saoirse Dolly, Mieke Van Hemelrijck

**Affiliations:** 1 Translational Oncology and Urology Research (TOUR), School of Cancer and Pharmaceutical Sciences, King’s College London, London, UK; 2 Department of Haematological Medicine, King’s College Hospital NHS Foundation Trust, London, UK; 3 King’s College Hospital NHS Foundation Trust, London, UK; 4 Medical Oncology, Guy’s and St Thomas’ NHS Foundation Trust (GSTT), London, UK; 5 School of Cancer and Pharmaceutical Sciences, King’s College London, London, UK; 6 Department of Haematological Medicine, Princess Royal University Hospital, Farnborough, UK; 7 Haematology Department, Guy’s and St Thomas’ NHS Foundation Trust (GSTT), London, UK; 8 Clinical Oncology, Guy’s and St Thomas NHS Foundation Trust (GSTT), London, UK; 9 Guy’s and St Thomas’ NHS Foundation Trust, London, UK; 10 Breast Cancer Genetics, King’s College London, London, UK

**Keywords:** Translational research, Risk factors, Oncology

## Abstract

**Background:**

Using an updated dataset with more patients and extended follow-up, we further established cancer patient characteristics associated with COVID-19 death.

**Methods:**

Data on all cancer patients with a positive reverse transcription-polymerase chain reaction swab for severe acute respiratory syndrome coronavirus-2 (SARS-CoV-2) at Guy’s Cancer Centre and King’s College Hospital between 29 February and 31 July 2020 was used. Cox proportional hazards regression was performed to identify which factors were associated with COVID-19 mortality.

**Results:**

Three hundred and six SARS-CoV-2-positive cancer patients were included. Seventy-one had mild/moderate and 29% had severe COVID-19. Seventy-two patients died of COVID-19 (24%), of whom 35 died <7 days. Male sex [hazard ratio (HR): 1.97 (95% confidence interval (CI): 1.15–3.38)], Asian ethnicity [3.42 (1. 59–7.35)], haematological cancer [2.03 (1.16–3.56)] and a cancer diagnosis for >2–5 years [2.81 (1.41–5.59)] or ≥5 years were associated with an increased mortality. Age >60 years and raised C-reactive protein (CRP) were also associated with COVID-19 death. Haematological cancer, a longer-established cancer diagnosis, dyspnoea at diagnosis and raised CRP were indicative of early COVID-19-related death in cancer patients (<7 days from diagnosis).

**Conclusions:**

Findings further substantiate evidence for increased risk of COVID-19 mortality for male and Asian cancer patients, and those with haematological malignancies or a cancer diagnosis >2 years. These factors should be accounted for when making clinical decisions for cancer patients.

## Introduction

To date, there have been over 33 million cases worldwide of infection with severe acute respiratory syndrome coronavirus-2 (SARS-CoV-2) that gives rise to COVID-19, with the number of deaths reaching over a million as of 1 September 2020 [1]. Considering the many undiagnosed infections and the lack of vital statistics for mortality in most countries, both these figures are serious underestimates. The COVID-19 pandemic has put an unprecedented strain on many healthcare systems, welfare states and economies. Understanding how COVID-19 affects cancer patients, including the clinical course of the disease, potential risk factors for severe COVID-19 and excess mortality in cancer patients, is essential for mitigation strategies, adaptation of site-specific models and pathways of care.

We previously reported results from 156 SARS-CoV-2-positive cancer patients from Guy’s Cancer Centre, London, identified between February and May 2020 [2]. With a median follow-up of 37 days, we found that 17.9% of cancer patients identified as positive for SARS-CoV-2 developed a severe infection, by World Health Organisation (WHO) classification [3], and 28.8% of this cohort died of COVID-19. Patients diagnosed with cancer for >2 years seemed to be at increased risk of severe COVID-19 disease compared to those diagnosed more recently. Furthermore, Asian ethnicity and being on palliative treatment were positively associated with an increased risk of death from COVID-19 in cancer patients. The true rate of COVID-19 disease in our oncology patients remains unquantified because the denominator is not known, i.e. the actual number of all cancer patients infected with SARS-CoV-2, as up to 80% of cases are mild or asymptomatic [4].

Since March 2020, there have been several cohort studies published presenting the clinical and demographic characteristics of cancer patients diagnosed with SARS-CoV-2 infection and/or their association with COVID-19 outcomes. Two recent systematic reviews summarised many of these studies, with the majority of data presented from China and Italy [5, 6]. Many of the studies identified subsets of cancer patients in a wider cohort, with few focusing on cancer patients only. Only a few studies with a case–control design have been reported [7–12]. Moreover, the outcomes investigated in the studies reviewed were very diverse. As a result, there were no clear conclusions as to what factors affects the course of COVID-19 disease in cancer patients specifically. Since these reviews were published, other multicentre observational studies have been published including that of Lee et al. [13]. That study included cancer patients from across the UK and concluded that mortality in cancer patients diagnosed with COVID-19 was mainly driven by age, sex and comorbidities [13]. Whilst this study included over 800 patients, there is a great level of heterogeneity between cancer centres across the UK, which can make it difficult to draw conclusions.

Herein, we present updated data on 306 COVID-19-positive cancer patients from Guy’s Cancer Centre and King’s College Hospitals NHS Foundation Trust. Both treat a diverse group of patients with a high proportion of ethnic and socio-economic variation due to their location within South East London. Together, they treat ~12,000 cancer patients each year and were at the epicentre of the first UK COVID-19 wave. By utilising data from two large centres with similar patient demographics and anti-cancer treatment approaches, we aim to draw meaningful conclusions to support the clinical decision-making for cancer patients diagnosed with COVID-19.

## Methods

### Study population

Data from Guy’s Cancer Centre and King’s College Hospital were utilised within this observational study. Guy’s Cancer Cohort is a research ethics committee-approved research database (Reference number: 18/NW/0297) of all routinely collected clinical data of cancer patients diagnosed or treated at Guy’s and St Thomas’ (GSTT) NHS Foundation Trust [14]. A material transfer agreement was in place to add routinely collected anonymised clinical data for cancer patients from King’s College Hospital—both Trusts are part of the same Academic Health Science Centre, King’s Health Partners. The dataset used in this study comprised demographic and clinical data on all cancer patients who had a positive polymerase chain reaction test for SARS-CoV-2 infection between 29 February and 31 July 2020. During this time ~2200 patients were tested for COVID-19. Until 30th April, patients were tested for SARS-CoV-2 if they were symptomatic and needed hospitalisation or if they were scheduled to undergo a cancer-related treatment. SARS-CoV-2 testing was then introduced as standard care thereafter. As previously described, we categorised COVID-19 based on the WHO criteria for disease severity [2]. However, in this analysis, we also included those who died from COVID-19 in the severe group.

### Statistical methods

This updated analysis was conducted according to the same three aims as our previous study [2]. In COVID-19 cancer patients, we aimed to:

1. describe their demographic and clinical characteristics,
2. determine if these were associated with COVID-19 severity
3. or COVID-19-related death.

The first aim was addressed using descriptive statistics. Socio-economic status (SES) (low, middle and high) was categorised based on the English Indices of Multiple Deprivation for postcodes [15]. Lymphocyte count (×10^9^) was defined as ≤0.5, 0.6–0.8, 0.9–1.2, and >1.2 based on the Common Terminology Criteria for Adverse Events v.5. For the other laboratory variables, we reported tertiles instead of clinical cut-offs due to cancer patients already having abnormal values for most of these blood markers (ferritin, C-reactive protein (CRP), and albumin). The radical treatment referred to those patients with a chance of long-term survival or cure.

We conducted logistic regression analyses to answer the second aim. Pneumonia with or without sepsis (i.e. those patients managed on the ward) was an indicator of mild/moderate COVID-19, whereas acute respiratory distress syndrome (ARDS) or septic shock (i.e. those patients where severity reaches criteria for Intensive Care Unit admission, if deemed clinically appropriate) were indicators of severe COVID-19—as defined by the WHO COVID-19 classification [3]. We used a directed acyclic graph (DAG) (Supplementary Fig. 1 in Appendix) to inform the models to quantify the association between each factor and COVID-19 severity. Each factor was individually set as the main exposure variable in the model when determining the minimal adjustments required (Supplementary Table 1 in Appendix).

We addressed the third aim with Cox proportional hazards regression analyses, whereby the models were defined as above (Supplementary Table 1 in Appendix). Follow-up was defined from the date of COVID testing until death or 31 July 2020. To further investigate COVID-19 fatality in cancer patients, we additionally looked at the risk of COVID-19 death within 7 days using Cox proportional hazards regression analysis. No clear guidelines are available about early death due to COVID-19 and hence the median time to death in patients who had died of COVID-19 (i.e. 7 days) was chosen as the cut-off. These multivariate analyses were also based on the DAG as described above [2] (Supplementary Fig. 1 in Appendix).

All statistical analyses were conducted with STATA version 15.1.

## Results

### Demographic and clinical characteristics of COVID-19-positive cancer patients

Of the 306 COVID-19-positive cancer patients, 218 (71%) had mild/moderate COVID-19 disease and 88 (29%) had severe COVID-19 as defined by the WHO criteria (Table 1). Most patients were male (60%) and aged >60 years (71%) (mean age: 66 years). When stratified by COVID-19 severity, there was a higher proportion of patients aged >60 years in the severe group (76% compared to the 68% in the mild/moderate group). There was a higher proportion of patients with high SES in the mild/moderate group (7%) compared to the severe group (2%). Fifty-seven per cent were White, 23% Black and 4% of Asian ethnicity. The most notable difference between the ethnicities was Asian ethnicity, in which 2% had mild/moderate and 9% had severe disease. With respect to comorbidities, 45% of patients had hypertension, 22% diabetes mellitus, 18% lung condition, 19% renal impairment and 17% cardiovascular disease and 70% had at least one comorbidity.

**Table 1:** Demographic characteristics of COVID-19-positive cancer patients.

	Total (*n* = 306)		WHO COVID grade			
			Mild/moderate (*n* = 218)		Severe (*n* = 88)	
	*n*	%	*n*	%	*n*	%
Sex						
Male	185	60.50	123	56.40	62	70.50
Female	121	39.50	95	43.60	26	29.50
Age						
<50	43	14.10	36	16.50	7	8.00
50–59	47	15.40	33	15.10	14	15.90
60–69	83	27.10	58	26.60	25	28.40
70–79	66	21.60	50	22.90	16	18.20
≥80	67	21.90	41	18.80	26	29.50
Mean (SD)	66.30 (15.24)		65.09 (15.31)		69.29 (14.70)	
SES						
Low	254	83.00	179	82.10	75	85.20
Medium	5	1.60	4	1.80	1	1.10
High	17	5.60	15	6.90	2	2.30
Missing	30	9.80	20	9.20	10	11.40
Ethnicity						
White British	152	49.70	108	49.50	44	50.00
White Other	22	7.20	15	6.90	7	8.00
Black Caribbean	23	7.50	14	6.40	9	10.20
Black Africa	25	8.20	20	9.20	5	5.70
Black Other	23	7.50	13	6.00	10	11.40
Asian	13	4.20	5	2.30	8	9.10
Mixed	2	0.70	1	0.50	1	1.10
Other	5	1.60	4	1.80	1	1.10
Unknown	41	13.40	38	17.40	3	3.40
Comorbidities						
Hypertension	137	44.80	96	44.00	41	46.60
Diabetes mellitus	67	21.90	46	21.10	21	23.90
Lung conditions	55	18.00	35	16.10	20	22.70
Renal impairment	59	19.30	40	18.30	19	21.60
Liver conditions	10	3.30	7	3.20	3	3.40
CVD	53	17.30	38	17.40	15	17.00
No. of comorbidities						
0	93	30.40	71	32.60	22	25.00
1	85	27.80	60	27.50	25	28.40
2	58	19.00	40	18.30	18	20.50
3+	70	22.90	47	21.60	23	26.10
Smoking history						
Never	128	41.80	91	41.70	37	42.00
Current	26	8.50	17	7.80	9	10.20
Ex-smoker	67	21.90	50	22.90	17	19.30
Unknown	85	27.80	60	27.50	25	28.40
Medications						
Polypharmacy	160	52.30	109	50.00	51	58.00
NSAIDs	36	11.80	24	11.00	12	13.60
ACE/ARB	79	25.80	50	22.90	29	33.00
Beta-blockers	57	18.60	39	17.90	18	20.50

The most common tumour type was haematological (although this likely reflects the large volume of haematological patients treated across both Trusts) (Table 2). The demographics of the haematology patients from the two Trusts were similar in terms of gender, SES and ethnicity. Of the haematological patients, 25 (21%) were myeloid and 92 (79%) were lymphoid malignancies. A third of the haematological patients had lymphoma, 11% chronic lymphocytic leukaemia (CLL), 13% acute leukaemia, 28% myeloma, 13% myelodysplastic syndrome/myeloproliferative neoplasm and 2% aplastic anaemia (Supplementary Table 2 in Appendix). The second most common cancer type was urological/gynaecological (20%), followed by breast (12%) and gastrointestinal (12%). Of 122 patients who acquired COVID-19 after a hospital admission, 78 (64%) had solid tumours and 44 (36%) haematological cancers. The majority of patients had advanced disease (stage IV, 51%), 33% were on palliative treatment and 27% on radical treatments. Of the 102 on palliative treatment, 48% were on first-line treatment, followed by 23% second line and 14% third line. Of 165 patients undergoing systemic anti-cancer therapy, the majority were on chemotherapy (72%). Notably, 43% of patients were >2 years from a primary cancer diagnosis. When stratified by COVID-19 severity, this proportion was 37% in those with mild/moderate disease and 59% for those with severe disease. There appeared to be a lower proportion of patients with performance status (PS) 0 in the severe group (7%) compared to the mild/moderate group (23%).

**Table 2:** Tumour characteristics of COVID-19-positive cancer patients.

	Total (*n* = 306)		WHO COVID grade			
			Mild/moderate (*n* = 218)		Severe (*n* = 88)	
	*n*	%	*n*	%	*n*	%
Cancer type						
Urological/gynae	61	19.90	47	21.60	14	15.90
Gastrointestinal	36	11.80	26	11.90	10	11.40
Haematological	117	38.20	71	32.60	46	52.30
Skin/head and neck/sarcoma	18	5.90	15	6.90	3	3.40
Central nervous system	13	4.20	10	4.60	3	3.40
Breast	38	12.40	35	16.10	3	3.40
Lung	23	7.50	14	6.40	9	10.20
Cancer stage						
I	33	10.80	28	12.80	5	5.70
II	45	14.70	38	17.40	7	8.00
III	39	12.70	31	14.20	8	9.10
IV	155	50.70	98	45.00	57	64.80
Missing	34	11.10	23	10.60	11	12.50
Risk category^a^ (*n* = 25)						
Low	11	44.00	4	33.30	7	53.90
Intermediate	5	20.00	4	33.30	1	7.70
High	8	32.00	4	33.30	4	32.00
Missing	1	4.00	0	0.00	1	7.70
Treatment paradigm						
Treatment naive	22	7.20	14	6.40	8	9.10
Neoadjuvant	13	4.20	13	6.00	0	0.00
Adjuvant	21	6.90	19	8.70	2	2.30
Radical	83	27.10	59	27.10	24	27.30
Palliative	102	33.30	70	32.10	32	36.40
Watch and wait	6	2.00	5	2.30	1	1.10
Surveillance	50	16.30	32	14.70	18	20.50
Missing	9	2.90	6	2.80	3	3.40
Line of palliative treatment (*n* = 99)						
1	49	49.50	33	48.50	16	51.60
2	23	23.20	16	23.50	7	22.60
3	14	14.10	11	16.20	3	9.70
4	1	1.00	1	1.50	0	0.00
Missing	12	12.10	7	10.30	5	16.20
Systemic treatment (*N* = 165)						
Systemic chemotherapy	119	72.12	88	70.97	31	75.61
Immunotherapy	9	5.45	6	4.84	3	7.32
Biological	19	11.52	17	13.71	2	4.88
Targeted therapy	6	3.64	4	3.23	2	4.88
Combination therapy	12	7.27	9	7.26	3	7.32
Time since cancer diagnosis						
<1 year	122	39.90	103	47.20	19	21.60
1–2 years	36	11.80	25	11.50	11	12.50
2–5 years	60	19.60	35	16.10	25	28.40
≥5 years	73	23.90	46	21.10	27	30.70
Missing	15	4.90	9	4.10	6	6.80
Performance status						
0	55	18.00	49	22.50	6	6.80
1	105	34.30	73	33.50	32	36.40
2	61	19.90	45	20.60	16	18.20
3	45	14.70	28	12.80	17	19.30
4	14	4.60	10	4.60	4	4.50
Missing	26	8.50	13	6.00	13	14.80

^a^For myeloid malignancies only.

The most common symptoms at presentation were: fever 68 vs 52%, cough 59 vs 47% and dyspnoea 66 vs 29% for those with severe vs mild/moderate disease (Table 3). Similarly, laboratory findings that were increased in severe disease included hyperferritinaemia ≥1596 in 25 vs 8%, raised CRP ≥126 in 53 vs 17%, lymphopenia ≤0.5 in 41 vs 23% and hypoalbuminaemia ≤32 in 49 vs 19%. Overall, 9% of patients were treated in the intensive treatment unit, increasing to 31% in the severe group.

**Table 3:** COVID-19 presentation of COVID-19-positive cancer patients.

	Total (*n* = 306)		WHO COVID grade			
			Mild/moderate (*n* = 218)		Severe (*n* = 88)	
	*n*	%	*n*	%	*n*	%
Symptoms						
Cough	143	46.70	91	41.70	52	59.10
Fever	158	51.60	98	45.00	60	68.20
Dyspnoea	121	39.50	63	28.90	58	65.90
Gastrointestinal symptoms	45	14.70	26	11.90	19	21.60
Time between first symptom and diagnosis						
<7 days	173	56.50	113	51.80	60	68.20
7–14 days	53	17.30	35	16.10	18	20.50
>14 days	21	9.60	7	8.00	28	9.20
Missing	52	17.00	49	22.50	3	3.40
Care setting						
Outpatient	68	22.20	67	30.70	1	1.10
Inpatient	210	68.60	150	68.80	60	68.20
ITU	27	8.80	0	0.00	27	30.70
Missing	1	0.30	1	0.50	0	0.00
Laboratory values*						
Ferritin (μg/L)						
T1 (80–793)	40	13.10	32	14.70	8	9.10
T2 (891–1442)	40	13.10	28	12.80	12	13.60
T3 (1596–5958)	39	12.70	17	7.80	22	25.00
Missing	187	61.10	141	64.70	46	52.30
CRP (mg/L)						
T1 (3–41)	86	28.10	75	34.40	11	12.50
T2 (42–117)	81	26.50	52	23.90	29	33.00
T3 (126–508)	83	27.10	36	16.50	47	53.40
Missing	56	18.30	55	25.20	1	1.10
Lymphocytes (×10^9^)						
≤0.5	87	28.40	51	23.40	36	40.90
0.6–0.8	70	22.90	47	21.60	23	26.10
0.9–1.2	51	16.70	40	18.30	11	12.50
>1.2	63	20.60	46	21.10	17	19.30
Missing	35	11.40	34	15.60	1	1.10
Albumin (g/L)						
T1 (20–32)	84	27.50	41	18.80	43	48.90
T2 (33–38)	98	32.00	65	29.80	33	37.50
T3 (39–57)	68	22.20	62	28.40	6	6.80
Missing	56	18.30	50	22.90	6	6.80

^a^Distribution shown in tertiles (T).

### Factors associated with COVID-19 severity in cancer patients

The overall rate of severe COVID-19 infection was 29%, 22% in solid tumours and 39% in haematological malignancies. Several factors were associated with an increased risk of severe COVID-19 disease (Table 4). These included male gender (odds ratio (OR): 1.84, 95% CI: 1.08–3.13), Asian ethnicity (OR: 3.86, 95% CI: 1.20–12.36), haematological cancer type (OR: 2.16, 95% CI: 1.18–3.95) and being diagnosed with cancer for 2–5 years (OR: 3.74, 95% CI: 1.80–7.78) or ≥5 years (OR: 3.06, 95% CI: 1.50–6.26). PS or treatment paradigm was not associated with severity. Symptomatic patients with cough (OR: 1.89, 95% CI: 1.12–3.19), fever (OR: 2.53, 95% CI: 1.46–4.38) and dyspnoea (OR: 5.04, 95% CI: 2.85–8.92) were at increased risk of severe COVID-19 disease compared to those without symptoms. The highest range of ferritin (1964–10,327 μg/L) was associated with severe disease (OR: 54.92, 95% CI: 5.90–511.33).

**Table 4:** Odds ratios and 95% confidence intervals for COVID-19 severity in cancer patients.

	OR^a^	95% CI
Sex		
Female	1.00	Ref.
Male	1.84	(1.08–3.13)
Age		
≤60	1.00	Ref.
>60	1.48	(0.84–2.61)
SES		
Low	1.00	Ref.
Middle	0.61	(0.06–5.76)
High	0.58	(0.12–2.87)
Ethnicity		
White	1.00	Ref.
Black	1.23	(0.68–2.22)
Asian	3.86	(1.20–12.36)
Other	0.96	(0.18–5.13)
Number of comorbidities		
0	1.00	Ref.
1	1.17	(0.55–2.48)
2	1.07	(0.46–2.47)
3+	0.98	(0.43–2.25)
*P* for trend	0.896	
Smoking history		
Never	1.00	Ref.
Ever	0.80	(0.41–1.57)
Cancer type		
Solid	1.00	Ref.
Haematological	2.16	(1.18–3.95)
Treatment paradigm		
No active treatment	1.00	Ref.
Radical/curative	1.46	(0.59–3.62)
Palliative	2.26	(0.94–5.43)
Time since cancer diagnosis		
<1 year	1.00	Ref.
1–2 years	2.30	(0.95–5.57)
2–5 years	3.74	(1.80–7.78)
≥5 years	3.06	(1.50–6.26)
Performance status		
0–2	1.00	Ref.
3+	1.21	(0.60–2.47)
Symptoms		
Cough	1.89	(1.12–3.19)
Fever	2.53	(1.46–4.38)
Dyspnoea	5.04	(2.85–8.92)
GI symptoms	1.86	(0.96–3.60)
Time between first symptom and diagnosis		
<7 days	1.00	Ref.
7–14 days	0.97	(0.51–1.85)
>14 days	0.63	(0.25–1.56)
Ferritin		
T1 (14–704)	1.00	Ref.
T2 (771–1962)	2.79	(0.47–16.64)
T3 (1964–10,327)	54.92	(5.90–511.33)
CRP (mg/L)		
T1 (1–50)	1.00	Ref.
T2 (51–145)	4.28	(1.57–11.66)
T3 (146–528)	7.38	(2.71–20.09)
Lymphocytes (×10^9^)		
≤0.5	1.00	Ref.
0.6–0.8	0.43	(0.17–1.07)
0.9–1.2	0.44	(0.16–1.20)
>1.2	0.44	(0.16–1.16)
Albumin (g/L)		
T1 (15–29)	1.00	Ref.
T2 (30–37)	0.62	(0.29–1.30)
T3 (38–109)	0.10	(0.03–0.35)

^a^Adjustment as defined by the DAG (Supplementary Table 1 in Appendix).

### Factors associated with COVID-19 death in cancer patients

The overall COVID-related mortality rate was 24%, 19% in solid and 32% in haematological cancers with a median follow-up of 134 days (interquartile range (IQR) 32–156). Similar patterns of associations were observed for the risk of COVID-19 death as for the severity of COVID-19 (Table 5). Factors that increased risk for COVID-related death were male sex (HR: 1.97, 95% CI: 1.15–3.38), Asian ethnicity (HR: 3.42, 95% CI: 1.59–7.35) and haematological cancer type (HR: 2.03, 95% CI: 1.16–3.56), as well as a cancer diagnosis for >2–5 years (HR: 2.81, 95% CI: 1.41–5.59) and ≥5 years (HR: 2.13, 95% CI: 1.06–4.27). In addition, age >60 years was also associated (HR: 2.14, 95% CI: 1.15–3.98) compared to age ≤60 years. Patients with fever and dyspnoea also had an increased risk of COVID-19 death compared to asymptomatic patients (HR: 1.86, 95% CI: 1.10–3.14 and HR: 3.34, 95% CI: 1.97–5.66, respectively). Again, ferritin in the highest tertile (HR: 16.11, 95% CI: 3.81–68.17) and highest two tertiles for CRP (HR: 2.88, 95% CI: 11.13–7.31 and HR: 4.10, 95% CI: 1.66–10.10) were at an increased risk of COVID-19 death compared to the lowest tertiles comparators. Meanwhile, those in the highest tertile for albumin had a decreased risk of COVID-19 death (HR: 0.12, 95% CI: 0.03–0.51).

**Table 5:** Hazard ratios and 95% confidence intervals for COVID-19 death in cancer patients.

Variable	Number of deaths (*n* = 72)	HR^a^	95% CI
Sex			
Female	19	1.00	Ref.
Male	53	1.97	(1.15–3.38)
Age			
≤60	13	1.00	Ref.
>60	59	2.14	(1.15–3.98)
SES			
Low	60	1.00	Ref.
Middle	1	0.89	(0.12–6.66)
High	2	0.83	(0.19–3.55)
Ethnicity			
White	40	1.00	Ref.
Black	19	1.21	(0.69–2.12)
Asian	8	3.42	(1.59–7.35)
Other	2	1.60	(0.39–6.65)
Number of comorbidities			
0	14	1.00	Ref.
1	22	1.50	(0.72–3.13)
2	16	1.40	(0.64–3.09)
3+	20	1.05	(0.48–2.31)
* P* for trend		0.887	
Smoking history			
Never	29	1.00	Ref.
Ever	21	0.85	(0.45–1.59)
Cancer type			
Solid	35	1.00	Ref.
Haematological	37	2.03	(1.16–3.56)
Treatment paradigm			
No active treatment	16	1.00	Ref.
Radical/curative	19	1.13	(0.50–2.52)
Palliative	28	1.95	(0.94–4.01)
Time since cancer diagnosis			
<1 year	16	1.00	Ref.
1–2 years	9	1.84	(0.79–4.30)
2–5 years	22	2.81	(1.41–5.59)
≥5 years	20	2.13	(1.06–4.27)
Performance status			
0–2	41	1.00	Ref.
3+	20	1.43	(0.78–2.62)
Symptoms			
Cough	41	1.38	(0.85–2.25)
Fever	47	1.86	(1.10–3.14)
Dyspnoea	47	3.34	(1.97–5.66)
GI symptoms	13	1.13	(0.60–2.12)
Time between first symptom and diagnosis			
<7 days	52	1.00	Ref.
7–14 days	12	0.64	(0.33–1.27)
>14 days	5	0.55	(0.22–1.37)
Ferritin			
T1 (14–704)	5	1.00	Ref.
T2 (771–1962)	7	2.91	(0.62–13.74)
T3 (1964–10,327)	18	16.11	(3.81–68.17)
CRP (mg/L)			
T1 (1–50)	9	1.00	Ref.
T2 (51–145)	23	2.88	(1.13–7.31)
T3 (146–528)	39	4.10	(1.66–10.10)
Lymphocytes (×10^9^)			
≤0.5	30	1.00	Ref.
0.6–0.8	19	0.68	(0.34–1.38)
0.9–1.2	10	0.68	(0.30–1.53)
>1.2	12	0.60	(0.25–1.42)
Albumin (g/L)			
T1 (15–29)	38	1.00	Ref.
T2 (30–37)	25	0.70	(0.39–1.24)
T3 (38–109)	4	0.12	(0.03–0.51)

^a^Adjustment as defined by the DAG (Supplementary Table 1 in Appendix).

### Factors associated with COVID-19 death within 7 days of diagnosis

Haematological cancer type (HR: 2.74, 95% CI: 1.21–6.22), 2–5 years since cancer diagnosis (HR: 4.81, 95% CI: 1.47–15.69), and >5 years since cancer diagnosis (HR: 4.41, 95% CI: 1.38–14.06) and patients who presented with dyspnoea (HR: 5.25, 95% CI: 2.14–12.89) were all associated with an increased risk of dying from COVID-19 within 7 days of diagnosis (Table 6). When looking at laboratory results, CRP levels in the highest tertile (146–528 mg/L) were also associated with an increased risk of an early death due to COVID-19 (HR: 12.70, 95% CI: 1.58–102.40) when compared to patients with CRP levels in the lowest tertile.

**Table 6:** Hazard ratios and 95% confidence intervals for COVID-19 death within 7 days of diagnosis in cancer patients.

Variable	Number of deaths within 7 days of COVID-19 diagnosis	HR^a^	95% CI
Sex			
Female	11	1.00	Ref.
Male	24	1.48	(0.70–3.13)
Age			
<60	6	1.00	Ref.
>60	29	2.32	(0.89–6.02)
SES			
Low	34	1.00	Ref.
Middle	0	N/A	N/A
High	0	N/A	N/A
Ethnicity			
White	18	1.00	Ref.
Black	11	1.61	(0.73–3.56)
Asian	3	2.64	(0.77–9.07)
Other	2	3.20	(0.74–13.94)
Number of comorbidities			
0	7	1.00	Ref.
1	10	1.20	(0.40–3.61)
2	10	1.53	(0.51–4.60)
3+	8	0.82	(0.25–2.68)
Smoking history			
Never	14	1.00	Ref.
Ever	12	1.24	(0.51–3.00)
Cancer type			
Solid	15	1.00	Ref.
Haematological	20	2.74	(1.21–6.22)
Treatment Paradigm			
No active treatment	10	1.00	Ref.
Radical/curative	7	0.66	(0.21–2.05)
Palliative	14	1.29	(0.49–3.38)
Time since cancer diagnosis			
<1 year	5	1.00	Ref.
1–2 years	4	2.90	(0.71–11.90)
2–5 years	12	4.81	(1.47–15.69)
5+ years	12	4.41	(1.38–14.06)
Performance status			
0–2	21	1.00	Ref.
3+	9	1.02	(0.40–2.62)
Symptoms			
Cough	22	1.73	(0.82–3.63)
Fever	20	1.33	(0.63–2.80)
Dyspnoea	26	5.25	(2.14–12.89)
GI symptoms	6	1.04	(0.39–2.71)
Time between first symptom and diagnosis			
<7 days	27	1.00	Ref.
7–14 days	5	0.38	(0.11–1.25)
>14 days	1	0.22	(0.03–1.63)
*Abnormal Laboratory*			
Ferritin			
T1 (14–704)	1	1.00	Ref.
T2 (771–1962)	3	N/A	N/A
T3 (1964–10,327)	6	N/A	N/A
CRP tertiles			
T1 (1–50)	2	1.00	Ref.
T2 (51–145)	10	0.62	(0.73-53.57)
T3 (146–528)	22	12.70	(1.58–102.40)
Lymphocytes			
≤0.5	13	1.00	Ref.
0.6–0.8	5	0.67	(0.21–2.15)
0.9–1.2	6	1.33	(0.41–4.29)
>1.2	10	1.20	(0.36–3.98)
Albumin			
T1 (15–29)	16	1.00	Ref.
T2 (30–37)	13	1.10	(0.47–2.58)
T3 (38–109)	3	0.23	(0.03–1.92)

N/A refers to HRs with too few outcomes to be calculated.

^a^Adjustment as defined by the DAG (Supplementary Table 1 in Appendix).

## Discussion

We present an updated analysis of our previous analyses of data from Guy’s Cancer Centre, with double the patient numbers and a longer follow-up of 21 weeks. Of 306 patients, 29% had severe disease, and COVID-related mortality was 24%. Male sex, Asian ethnicity, haematological cancer, ≥24 months since cancer diagnosis, fever, dyspnoea, highest range ferritin and CRP were all associated with an increased risk of severe infection and COVID-19-specific death in cancer patients. In addition, those aged >60 years were at a higher risk of COVID-19-associated death compared to those aged 60 years or below. These findings are concordant with our preliminary analysis of 156 cancer patients [2]. From all the factors identified in our COVID-19-related death analysis, only haematological cancer type, a longer-established cancer diagnosis (2–5 years and >5 years), dyspnoea at the time of diagnosis and high levels of CRP were indicative of an early COVID-19-related death (within 7 days of diagnosis) in cancer patients. Our updated results strengthen the observations from the first analysis, with more statistical power and highlight the reproducibility of the data.

### Demographic and clinical characteristics

The median age of 66 years at COVID-19 diagnosis observed in the current cohort is similar to that of other studies, e.g. 63 years in both Liang et al. [16] and Yang et al. [17] and 65 in Zhang et al. [18]. Our distribution of male to female ratio (40% female) is similar to other large cohorts, including that of Guan et al. (42% female) [19]. In terms of ethnicity, our data are reflective of the cancer population seen by our Cancer Network. Nevertheless, it may be important to note that our study differed from that of Niedzwiedz et al. [20], in which 95% white ethnicity was reported for a non-cancer COVID-19 patient population from a similar area in South London, compared to only 57% of patients in our cohort [20]. The diverse ethnic makeup of our cohort has allowed us to observe the higher mortality associated with Asian ethnicity in particular.

In our study, there was a relatively high proportion of patients with good Eastern Cooperative Oncology Group (ECOG) PS of 0–2 (72%). One explanation for this is that this reflects the number of patients undergoing radical or active treatment undertaken at Guy’s Cancer Centre. This proportion is comparable to that reported by Kuderer et al. (75%) [21] and is slightly lower than Yang et al. (94%) [17]. As the pandemic has advanced, like many cancer centres, we have introduced increased screening of ambulant patients in an attempt to control infection transmission in the hospital setting, and this is likely to have increased the proportion of patients with good PS in our cohort.

### Risk of severe COVID-19 and COVID-19 death

There is a high level of alignment between the positive associations observed for risk of severe infection and COVID-19-related death. Whilst this trend was seen previously, it is likely the larger sample size has helped improve the statistical power in the new analysis. Furthermore, our findings suggest that from all the factors identified with COVID-19-related death, only haematological cancer type, a longer-established cancer diagnosis (2–5 years and >5 years), dyspnoea at the time of diagnosis and high levels of CRP were indicative of an early COVID-19-related death (within 7 days of diagnosis) in cancer patients.

Our study found that men were found to be at a significantly increased risk of severe infection and COVID-19-related death. This appears to be a frequently observed association for the general population, not only for cancer patients [22, 23]. One rationale for this link is the *TMPRSS2* gene, which is upregulated by androgens, and the protein product of which is used by the virus for cell entry [22, 23]. This might suggest a protective effect in prostate cancer patients on anti-androgens, but there was no clear association in our data or the literature [23].

Ethnicity has frequently been observed as a prognostic factor for COVID-19 patients, specifically for those of black, Asian and minority ethnic (BAME) groups [24]. In the current study, Asian ethnicity was found to increase the risk of both severe COVID-19 and death. Other studies have additionally highlighted an increased risk of COVID-19 death for patients of black ethnicity [24], an observation not reported within this study. Nevertheless, data from a similar South London non-cancer population also found an increased risk of in-hospital mortality for Asian ethnicity only [25]. The same study did also report an increased admission risk for those of both black and mixed/other ethnicities [25].

We found an increased risk of COVID-19 severity and death (including within 7 days) for haematological patients compared to those with solid malignancies. This is likely explained by the intense immunosuppressive treatment received by these patients, and by the fact that those who have undergone a transplant will be hospitalised. A deeper dive into the treatments received by the patients was carried out and is described in more detail in the Appendix (Supplementary Table 3). With regards to stem cell transplants (STCs), four patients received allogenic STCs, no patients underwent autogenic SCT as their most recent line of treatment, whilst four patients underwent autogenic SCT at any stage of their treatment. During the first wave of the pandemic, there was a hiatus in autogenic SCTs, which is reflected in our numbers. In addition, the haematological disease itself can cause immunosuppression with severe disease observed in patients who had never received treatment such as asymptomatic CLL as part of standard care. Other evidence suggests that haematological cancer patients are at an increased risk of COVID-19 compared to the general population [26, 27], but a study by El-Sharkawi and Iyengaret al. stated that there is a paucity of studies comparing them to solid malignancies [28].

There have been mixed reports as to whether PS is a prognostic factor for cancer patients with COVID-19. In this study, PS was not found to be associated with either risk of severe COVID-19 or death. A similar result was seen in a large cohort study including both solid and haematological cancer patients [17]. However, another study investigating only patients with haematological malignancies found an association between PS and survival (although only in univariate analysis, *p* < 0.0001) [29]. Furthermore, the risk of 30-day mortality was reportedly increased in cancer patients with a PS of two or more (when compared to 0 or 1) in a study from the USA (OR: 3.89, 95% CI: 2.11–7.18) [21].

Patients diagnosed with cancer >2 years ago (both 2–5 years and ≥5 years) were at an increased risk of severe COVID-19, death and early death within 7 days of diagnosis. This result might be reflective of more advanced disease status and in turn more likely to be associated with ongoing palliative systemic treatment. Of those on a palliative treatment intent, the majority were on at least second-line therapy. On the contrary, those diagnosed for >5 years may also represent patients who are either cured or are performing very well, although patients were only included in the analysis if they had undergone cancer treatment in the past 2 years. To the best of our knowledge, no other studies have investigated this as a prognostic factor in COVID-19-positive cancer patients.

High ferritin levels were found to be highly associated with an increased risk of both severe COVID-19 and death when compared to those with low ferritin. Very high serum ferritin levels have previously been observed in COVID-19 patients [30]. Furthermore, the association between raised CRP and increased risk of COVID-19 severity and death observed in this study has also been observed in other studies [31, 32], as might be expected for a biomarker associated with the severity of infectious and inflammatory diseases in general. It is worth noting that CRP levels are more likely to be measured in unwell patients (99% had CRP levels with severe disease) compared to those with mild–moderate disease (75%). Whilst raised CRP and hypoalbuminaemia occur commonly in cancer patients, it may limit the usefulness as a prognostication tool. Although, in the CORONET study, high CRP levels were considered the most important feature in predicting COVID-19 severity [33].

### Strengths and limitations

This study utilises data from over 300 SARS-Cov-2-positive cancer patients, with details of their infection and cancer at an individual level. Data from two large central London Cancer Centres allows analysis of cancer patients with diverse demographics, but with common treatment plans. We were, however, only able to report outcomes for cancer patients who were known to be SARS-CoV-2 positive. This ascertainment bias is born out of the challenges with testing all cancer patients even at the height of the pandemic as described in our previous study [34].

We report real-world data on a diverse London population for patients receiving all forms of cancer treatments. These data were validated by consultant cancer physicians. Despite validation, there are missing data for variables including smoking status (28%) and ethnicity (13%). It is also likely that some cancer patients with documented SARS-Cov-2 infection were missed from the analysis, if they received a positive SARS-CoV-2 test at another hospital, in the community or in a private setting. We tried to minimise this through links with external hospitals, but would have missed cases tested outside our geographic network or in the private sector.

We present extended follow-up data in the current analysis with a median follow-up of 134 days, compared to 37 days previously, which is substantially longer than most previous studies of ~4–8 weeks. A further advantage to the study is the use of a DAG to determine the appropriate variables to adjust for within the multivariate analyses. This is not something that has been observed in other COVID-19 studies to date. As highlighted by Wynants et al. [35], there is a paucity of controls within the studies investigating the intersection between COVID-19 and cancer, and therefore this is an area of interest for future studies.

## Conclusion

This study, with extended follow-up, re-iterates the increased risk of severe COVID-19 infection and related death for cancer patients of the male gender, Asian ethnicity, haematological malignancies and those diagnosed with cancer for >2 years. These risk factors should be taken into account in the clinical management of these patients during the pandemic. A core strength of this study is the use of data from two large clinical centres, with homogeneous care plans from within the same Academic Health Science Centre. As a result, we have been able to explore the intersection of COVID-19 and cancer in terms of clinical outcomes on one of the largest single network series to date.

## Supplementary information

Reproducibility checklist

Appendix

Guy’s Cancer Real World Evidence Authors

## Data Availability

Data can be obtained by researchers via an application to the Access Committee of Guy’s Cancer Cohort. An application form can be obtained via cancerdata@gstt.nhs.uk.
