# Supplementary material for: Risk of COVID-19 death in cancer patients: an analysis from Guy’s Cancer Centre and King’s College Hospital in London
Source: Br J Cancer. 2021 Aug 16;125(7):939–47. doi: 10.1038/s41416-021-01500-z (PMC8366163; doi:10.1038/s41416-021-01500-z)
Supplement: Supplementary file 2 — Appendix [file 41416_2021_1500_MOESM2_ESM.docx]

**Appendix**

**Supplementary Figure 1.** Directed Acyclic Graph (DAG) for the association between demographic and clinical characteristics of COVID-19 positive cancer patients and severity/death of COVID-19 (www.dagitty.net).


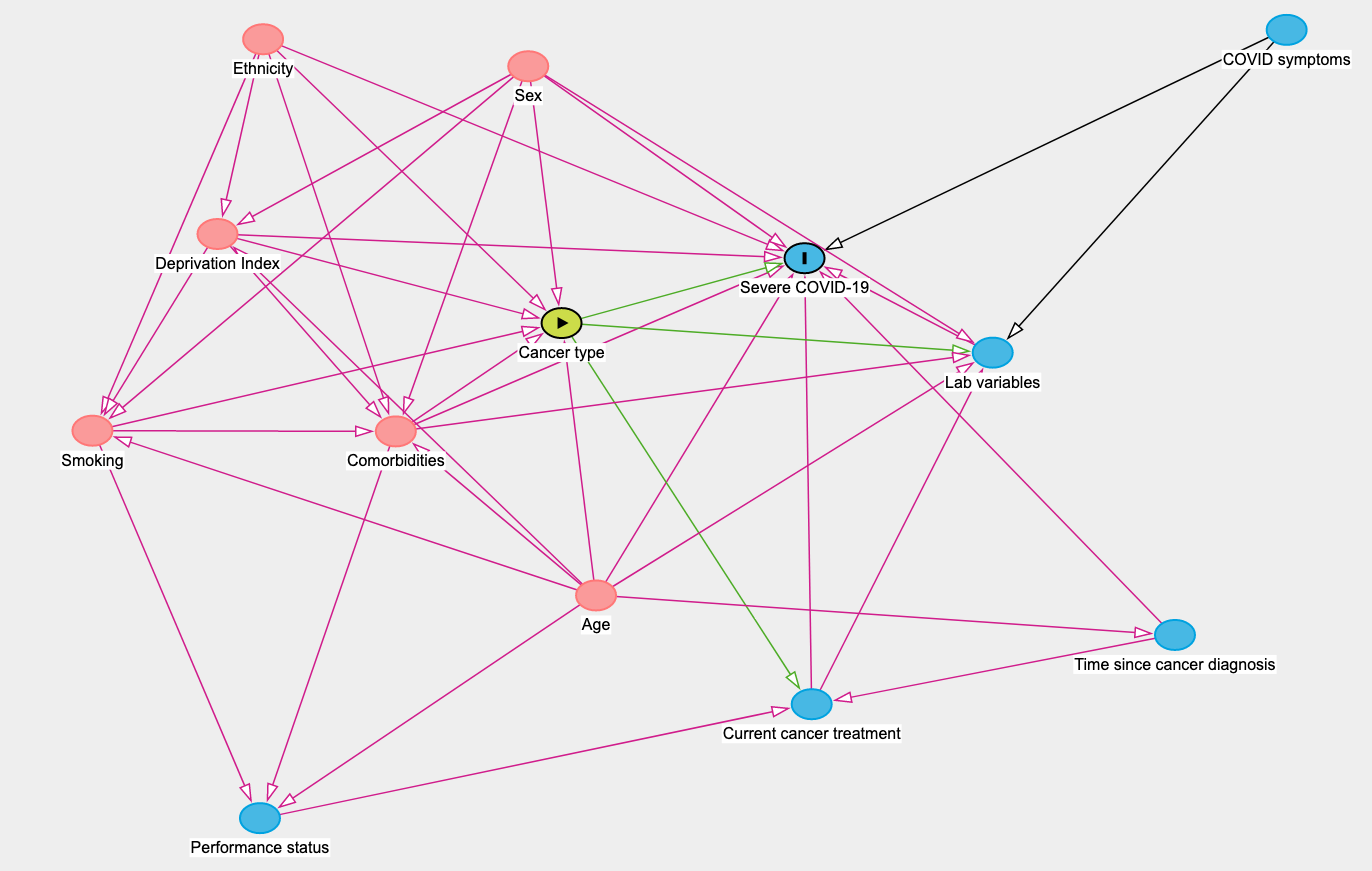


**Supplementary Table 1.** Overview of minimal adjustments for the associations between demographic and clinical characteristics of COVID-19 positive cancer patients and severity/death of COVID-19 (www.dagitty.net).

| **Main exposure variable** | **Minimal adjustments** |
| --- | --- |
| Age | No adjustment is necessary to estimate the total effect of Age on severity/death of COVID-19 |
| Sex | No adjustment is necessary to estimate the total effect of Sex on severity/death of COVID-19 |
| Ethnicity | No adjustment is necessary to estimate the total effect of Ethnicity on severity/death of COVID-19 |
| Deprivation Index | Age, Ethnicity, Sex |
| Comorbidities | Age, Deprivation Index, Ethnicity, Sex, Smoking |
| Smoking | Age, Deprivation Index, Ethnicity, Sex |
| Cancer type | - Age, Comorbidities, Deprivation Index, Ethnicity, Performance status, Sex - Age, Comorbidities, Deprivation Index, Ethnicity, Sex, Smoking |
| Current cancer treatment | - Age, Cancer type, Comorbidities, Deprivation Index, Ethnicity, Sex, Time since cancer diagnosis - Age, Cancer type, Comorbidities, Smoking, Time since cancer diagnosis - Cancer type, Performance status, Time since cancer diagnosis |
| Time since cancer diagnosis | Age |
| Performance status | Age, Cancer type, Comorbidities, Deprivation Index, Ethnicity, Sex  Age, Comorbidities, Smoking |
| Laboratory variables | Age, COVID symptoms, Cancer type, Comorbidities, Current cancer treatment, Sex |

**Supplementary Table 2.** Breakdown of haematological malignancies

| **Cancer Subgroup**  **(Total n=117)** | **N** | **%** |
| --- | --- | --- |
| Lymphoma | 39 | 33.30% |
| CLL | 13 | 11.10% |
| Symptomatic myeloma | 16 | 13.70% |
| Asymptomatic myeloma / MGUS | 17 | 14.50% |
| MDS / MDS-MPN / MPN | 15 | 12.80% |
| Acute leukaemia | 15 | 12.80% |
| Aplastic anaemia | 2 | 1.70% |

*MGUS - monoclonal gammopathy of unknown significance; MDS - Myelodysplastic syndromes; MPN - Myeloproliferative neoplasm*

**Supplementary Table 3.** Breakdown of treatment types for haematological patients

| **Treatment group**  **(Total n=117)** | N | % |
| --- | --- | --- |
| “Active monitoring” (watch and wait) | 36 | 30.80% |
| Anti-CD20 mAb containing regimens | 24 | 20.50% |
| Intensive highly myelosuppressive regimens | 13 | 11.10% |
| Combination conventional chemotherapy | 7 | 6.00% |
| Imid based | 9 | 7.70% |
| Transplant - Allogeneic HSC | 4 | 3.40% |
| Velcade based | 7 | 6.00% |
| Other | 17 | 14.50% |
