# Supplementary material for: Risk of COVID-19 death in cancer patients: an analysis from Guy’s Cancer Centre and King’s College Hospital in London
Source: Br J Cancer. 2021 Aug 16;125(7):939–47. doi: 10.1038/s41416-021-01500-z (PMC8366163; doi:10.1038/s41416-021-01500-z)
Supplement: Supplementary file 3 — Guy’s Cancer Real World Evidence Authors [file 41416_2021_1500_MOESM3_ESM.docx]

**Authors to include in Guy’s Cancer Real World Evidence**

Eleanor Jones

[Eleanor.Jones@gstt.nhs.uk](mailto:Eleanor.Jones@gstt.nhs.uk)

Pavetha Seeva

[Pavetha.Seeva@gstt.nhs.uk](mailto:Pavetha.Seeva@gstt.nhs.uk)

Christina Karampera

[Christina.Karampera@gstt.nhs.uk](mailto:Christina.Karampera@gstt.nhs.uk)

Aarani Devi

[Aarani.Devi@gstt.nhs.uk](mailto:Aarani.Devi@gstt.nhs.uk)

Fareen Rahman

[Fareen.Rahman@gstt.nhs.uk](mailto:Fareen.Rahman@gstt.nhs.uk)

Daniel Smith

[Daniel.Smith@gstt.nhs.uk](mailto:Daniel.Smith@gstt.nhs.uk)

Kasia Owczarczyk

[Kasia.Owczarczyk@gstt.nhs.uk](mailto:Kasia.Owczarczyk@gstt.nhs.uk)

Eirini Tsotra

[Eirini.Tsotra@gstt.nhs.uk](mailto:Eirini.Tsotra@gstt.nhs.uk)

Charalampos Gousis

[Harris.Gousis@gstt.nhs.uk](mailto:Harris.Gousis@gstt.nhs.uk)

Mary Lei

[Mary.Lei@gstt.nhs.uk](mailto:Mary.Lei@gstt.nhs.uk)

Sharmistha Ghosh

[Sharmistha.Ghosh@gstt.nhs.uk](mailto:Sharmistha.Ghosh@gstt.nhs.uk)

George Nintos

[George.Nintos@gstt.nhs.uk](mailto:George.Nintos@gstt.nhs.uk)

Kavita Raj

[Kavita.Raj@gstt.nhs.uk](mailto:Kavita.Raj@gstt.nhs.uk)

Mary Gleeson

[Mary.Gleeson@gstt.nhs.uk](mailto:Mary.Gleeson@gstt.nhs.uk)

Katherine Bailey

[Kate.Bailey@gstt.nhs.uk](mailto:Kate.Bailey@gstt.nhs.uk)

Richard Dillon

[Richard.Dillon@gstt.nhs.uk](mailto:Richard.Dillon@gstt.nhs.uk)

Matthew Streetly

[Matthew.Streetly@gstt.nhs.uk](mailto:Matthew.Streetly@gstt.nhs.uk)

Anca Mera

[anca.mera@kcl.ac.uk](mailto:anca.mera@kcl.ac.uk)

Jasmine Timbres

[jasmine.timbres@kcl.ac.uk](mailto:jasmine.timbres@kcl.ac.uk)
